# RNA cargos in extracellular vesicles derived from blood serum in pancreas associated conditions

**DOI:** 10.1038/s41598-020-59523-0

**Published:** 2020-02-18

**Authors:** Senthil R. Kumar, Eric T. Kimchi, Yariswamy Manjunath, Saivaroon Gajagowni, Alexei J. Stuckel, Jussuf T. Kaifi

**Affiliations:** 1 Veterinary Medicine & Surgery, College of Veterinary Medicine, University of Missouri, Columbia, MO 65211 USA; 2 Department of Surgery, School of Medicine, University of Missouri, Columbia, MO 65212 USA; 3 Harry S. Truman Veterans Hospital, 800 Hospital Drive, Columbia, MO 65212 USA; 4 Department of Medicine, Division of Gastroenterology and Hepatology; University of Missouri, Columbia, MO 65212 USA

**Keywords:** Gastrointestinal diseases, Oncology

## Abstract

Exosomes are extracellular vesicles which are released from healthy and tumor cells into blood circulation. Unique biomolecular cargos such as RNA and protein are loaded in these vesicles. These molecules may have biological functions such as signaling, cell communications and have the potential to be analyzed as biomarkers. In this initial study, we describe the analysis of exosomes in the serum of healthy subjects, intraductal papillary mucosal neoplasms and pancreatic ductal adenocarcinoma including the characterization of their RNA cargos by next generation sequencing (EXO-NGS). Results indicate the presence of a wide variety of RNAs including mRNA, miRNA, lincRNA, tRNA and piRNA in these vesicles. Based on the differential mRNA expression observed upon EXO-NGS analysis, we independently evaluated two protein coding genes, matrix metalloproteinase-8 (*MMP-8*) and transcription factor T-Box 3 (*TBX3*) by qRT-PCR for selective expression in the serum samples. Results indicate a variable expression pattern of these genes across serum samples between different study groups. Further, qRT-PCR analysis with the same serum exosomes processed for EXO-NGS, we observed two long non-coding RNAs, *malat-1* and *CRNDE* to be variably expressed. Overall, our observations emphasize the potential value of different exosome components in distinguishing between healthy, premalignant and malignant conditions related to the pancreas.

## Introduction

Currently, only a small number of useful biomarkers for the clinical diagnosis of different types of cancer. These have been used for several years without any considerable changes. This results in patients undergoing unnecessary medical procedures. This is further compounded by the fact that a single cancer type may be heterogeneous, presumably due to disparate genetic defects in their tumors^1^. Recently, studies have been directed towards identification of biomarkers for cancer and other diseases, through non-invasive means utilizing components in blood such as circulating tumor cells (CTCs), cell free DNA (cfDNA) and very recently extracellular vesicles (EVs) which includes microvesicles and exosomes^2–4^. These liquid biopsy (noninvasive) based analysis present an alternate to conventional tumor biopsies (invasive) and may facilitate biomarker identification to detect early stages of a disease.

EVs such as exosomes are released into blood circulation and may have important functions in physiological and pathological conditions. These vesicles have recently invoked interest due to their potential in disease diagnosis and treatment^3,4^. They originate from a multivesicular body and upon fusing with the plasma membrane of the cell, they are released into the extracellular environment^5^. Exosomes range between 30–150 nm in size, are secreted by different cell types including tumor cells^6,7^ and are heterogeneous which could reflect the phenotypic state of the cell that generates them^8^. The presence of exosomes has been reported in different biological fluids such as saliva, semen, urine, cerebrospinal fluid, breast milk and blood^9–12^. Due to the presence of exosomes in variety of pathological conditions, it has been investigated as a source of novel biomarkers based on analysis of their protein and RNA content. Nucleic acids such as mRNA, miRNA, transfer RNAs and long non-coding RNAs (lncRNAs) have been detected in exosomes^13,14^. Further, nucleic acid inside exosomes could be more resistant to RNA degrading enzymes such as RNases compared to those present in free circulation, which allows for a higher detection sensitivity and specificity^12^. The utility of exosomes and their cargos, especially the protein content has been previously reported for early detection of pancreatic cancer and predicting organotropic metastasis^15,16^. However, the RNA elements in exosomes and their probable utility as biomarkers are less explored in this disease.

PDAC is projected to become the second leading cause of cancer-related mortality in the United States by 2030^17^. Due to the lack of screening imaging modalities and specific biomarkers, early diagnosis of this disease is difficult and about 50% of all patients are diagnosed at advanced stages^18^ and have limited therapeutic options. Unique challenges associated with PDAC include aggressive etiology and deep anatomic location. Currently, serum C19-9 is used as a circulating biomarker for PDAC, although its sensitivity and specificity are less than enough for its use as a diagnostic or early stage biomarker^3^. Recently, exosome associated protein glypican has been reported as an early detection marker for PDAC^15^. However, this was disputed by different studies which indicated that glypican may not be an ideal marker to detect early PDAC^19,20^ because of its presence in non-malignant conditions.

Profiling of RNAs in circulation, specifically miRNAs, has been reported to identify biomarkers for pathologies including cancer^21–23^. A majority of these RNAs appears to be associated with exosomes. In this initial study, our underlying objective was the isolation and characterization of exosomes in blood serum along with the analysis of RNA cargos present in these vesicles by next generation sequencing (EXO-NGS). Further, we wanted to evaluate any variation in gene expression identified by EXO-NGS across healthy subjects, premalignant and malignant conditions associated with the pancreas. Such comparisons would offer a minimally invasive means of screening exosome RNA biomarkers that lay the groundwork in identifying potential biomarkers for early disease detection.

## Results and Discussion

### Isolation and characterization of serum exosomes

**Figure 1:**
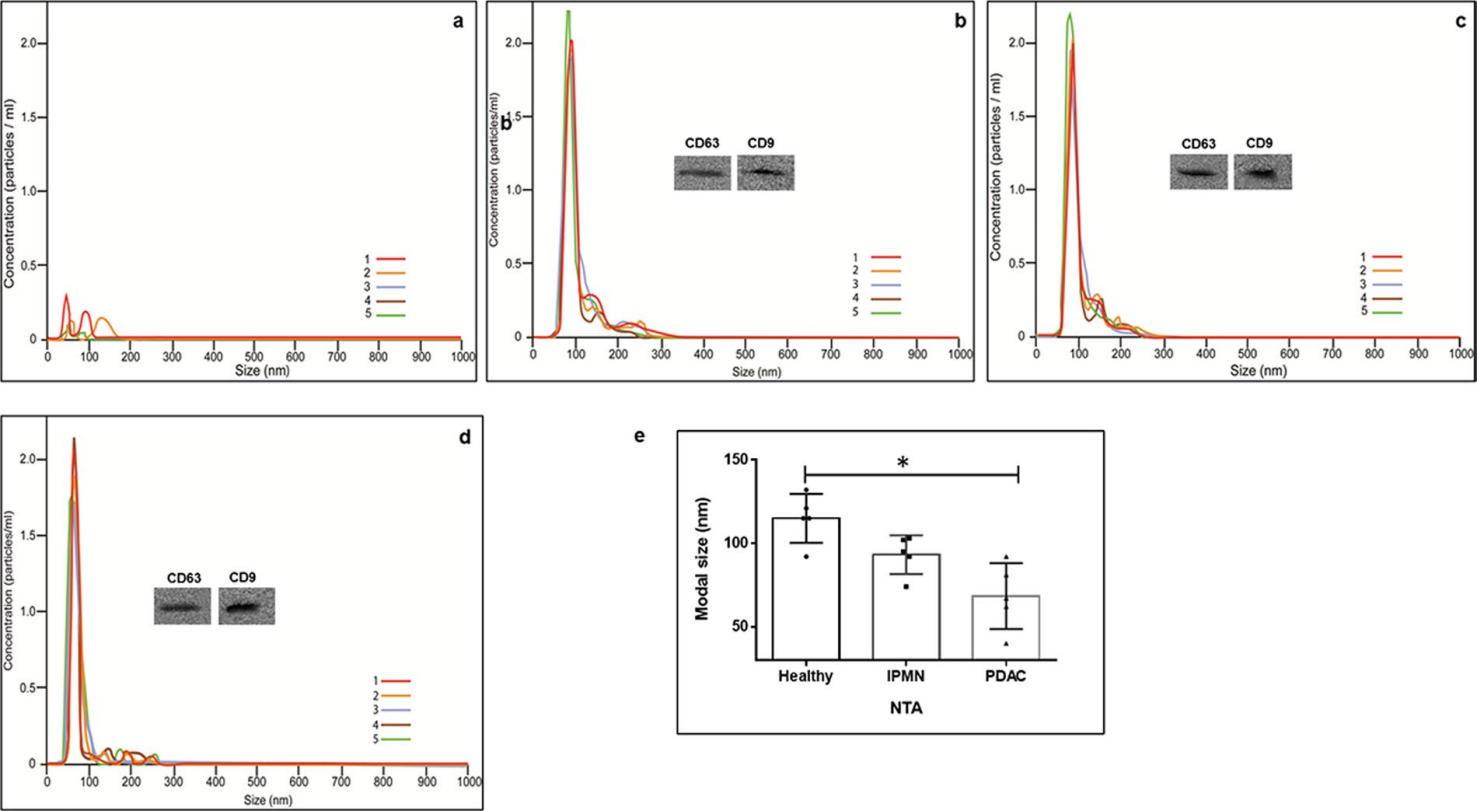
Isolation and characterization of exosomes from healthy, IPMN and PC serum. Exosomes from serum were separated using Exoquick^TC^ and purified further using gel exclusion chromatography as mentioned in methods. Exosome size was measured using Nanosight NS300 (Malvern Panalytical, Malvern, UK). (**a**) Buffer only; (**b**) Healthy serum; (**c**) IPMN serum; (**d**) PDAC serum; Five replicates were performed and modal size distribution was analyzed to assess significance in size differences. (**e**) Figure depicting the modal size of exosomes in the serum. Size differences between healthy and PDAC serum exosomes was significant (*p < 0.05). Immunoblot analysis were done to identify protein markers such as CD63 and CD9 with respective antibodies. Immunoblots are cropped images from different gels. Uncropped images are shown in Supplementary Fig. 1.

Several commercial kits are available to isolate exosomes from biological fluids which typically consists of polymeric additives to induce precipitation of exosomes^24^. Use of these kits along with a standard centrifugation process (10,000 × g) allows for a faster separation of exosomes compared to the gold-standard technique of ultracentrifugation which is both laborious and time-consuming. Moreover, studies have reported that the yields and purity using these kits were higher compared to ultracentrifugation^25,26^. While several options are available to isolate exosomes, depending on the source of exosomes and the purpose of the study, the protocol needs to be optimized to obtain a reasonably high yield and purity of these vesicles for downstream applications. We obtained exosomes (Fig. 1) from different serum samples within the size range (30–150 nm) which is in keeping with the previous studies^27^.

**Figure 2:**
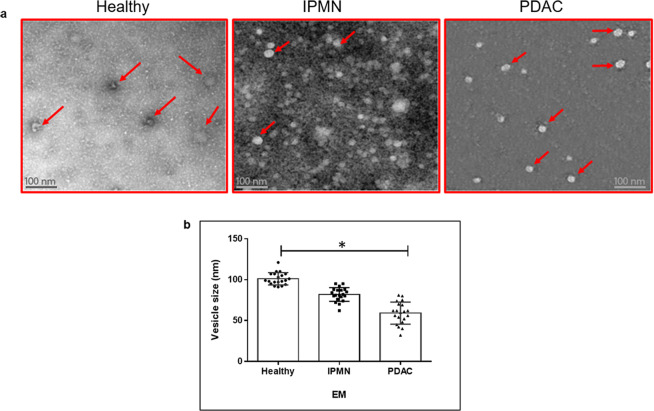
Ultrastructure analysis of exosomes in healthy, IPMN and PDAC serum by TEM. Exosomes were separated from serum samples using Exoquick^TC^, and subsequently purified by gel exclusion chromatography mentioned in methods. The samples were prepared for TEM and negative staining (n = 3 each). Images were acquired using JEOL JEM 1400 at 120 kV on a Gatan Ultrascan 1000 CCD. Red arrows depict exosomes. (**a**) Exosome vesicles in serum samples (n = 3 each) from healthy, IPMN and PDAC were measured (twenty vesicles/sample) randomly for their size distribution, and the median values are depicted. Vesicle size were lower in PDAC compared to healthy serum samples (*p = 0.032) (**b**).

In our study, exosomes isolated from the serum of healthy subjects and different pancreatic conditions were analyzed for their size distribution and quantity using Nanosight NS300 nanoparticle tracking analysis (NTA). Instruments such as dynamic light scattering and NTA have been used previously to measure the size of these vesicles^28^. NTA instrument in addition to size distribution also measures the nanoparticle concentrations^29^. The size distribution of exosomes analyzed by NTA between serum samples were different. The normal serum exosomes were predominantly 115 nm ± 20 nm in size while IPMN and PDAC serum exosomes exhibited sizes in the range of 94 nm ± 10 nm and 67 nm ± 25 nm, respectively (Fig. 1). While NTA was useful in analyzing exosome size and concentration the technique may have drawbacks. For instance, the size distributions obtained with this method may depend on set-up-parameters, and the concentration measurements based on refractive index of the material under examination. Further, underestimation of small size particles may be observed due to material-dependent lower detection limit. Hence more than one method may be necessary to analyze exosome morphology and size distribution. Further examination of purified vesicles by transmission electron microscopy (TEM) revealed that these vesicles possess varying size (35–100 nm) and morphology (Fig. 2a) resembling that of exosomes. The average size of serum exosomes as observed by EM was found to be 100 nm (range 90–120 nm) in healthy, 85 nm (range 62–95 nm) in IPMN and 59 nm (range 32–75 nm) in PDAC (Fig. 2b). There appears to be no consensus with regards to the minimal or maximal size range of exosomes in the literature. The lower size range reported varied between 30–50 nm and the upper size range varied between 100–150 nm^24,30^. This discrepancy could be due to the various exosome isolation methods employed during the studies as well as their source of origin. Our own studies indicated that exosomes isolated from cell culture supernatants tend to be of relatively larger size (~125 nm) compared to liquid biopsy samples such as serum but well within the upper range of 150 nm (data not shown). Collectively, in our current study we observed that serum exosomes appear to be in the size range of 32–130 nm.

The concentration of exosomes was found to be different in the serum of healthy subjects (7 × 10^8^ particles/ml), patients with IPMN (7.06 × 10^10^ particles/ml) and patients diagnosed with PDAC (4 × 10^11^ particles/ml). Exosome concentrations in the serum from PDAC patients tend to be higher than healthy or IPMN samples. Previous reports indicate that exosome concentrations can vary between 1–3 × 10^12^ particles per ml serum^27,31^. To confirm the vesicles were indeed exosomes, protein markers for exosomes such as CD63 and CD9 were analyzed using immunoblot studies. Serum exosomes from different conditions exhibit both the presence of CD63 and CD9 (Fig. 1 inset), widely reported to function as exosome markers^32,33^. Uncropped gel images are shown in supplementary (Fig. S1).

### RNA recovery from exosomes

Total RNA was isolated from different serum exosomes and the RNAs were purified using a phenol-free lysis buffer and rapid spin columns. RNA from serum exosomes isolated from each sample category was verified for integrity using a Bioanalyzer. A representative analysis of the small RNAs in serum samples are shown in supplementary (Fig. S2). Exosomes derived from serum typically yielded total RNA in the range of 2.4–12 ng/ml. RNA concentrations up to 10 ng were reported earlier in exosomes^27^. However, depending on the source of the starting material and the concentration of exosomes, the RNA concentrations could vary. Moreover, exosomes are a heterogeneous population of vesicles with widely varying cargo, in that some vesicles may have many RNAs while some carry only the protein cargo and no RNA.

### Analysis of RNA in exosomes by sequencing

Two donors were used for each sample type. The library construction was made by ligating the adaptor sequences to the RNA in the samples. Subsequently, PCR amplification was performed^34^. Once the library construction was complete, the samples were subjected to sequencing using the NextSeq. 500/550 instrument in high output configuration. The adaptor sequences were trimmed after sequencing exosome libraries and single-end RNA reads were generated. An example of the number of reads pre and post quality filtering is depicted in supplementary (Fig. S3) for different samples. The total number of reads in millions (M) retained after quality assessment were approximately 10 M for healthy serum, 25 M for IPMN and 19 M for PDAC. Further, the reads retained or discarded after quality assessment is depicted in supplementary (Fig. S4). About 75–80% of the reads in different serum samples were of good quality reads. Our analysis also included spike-in-DNA controls^35^ to avoid any misinterpretations during data normalization, based on the presumption that different serum samples will not yield identical amounts of RNA for analysis.

**Figure 3:**
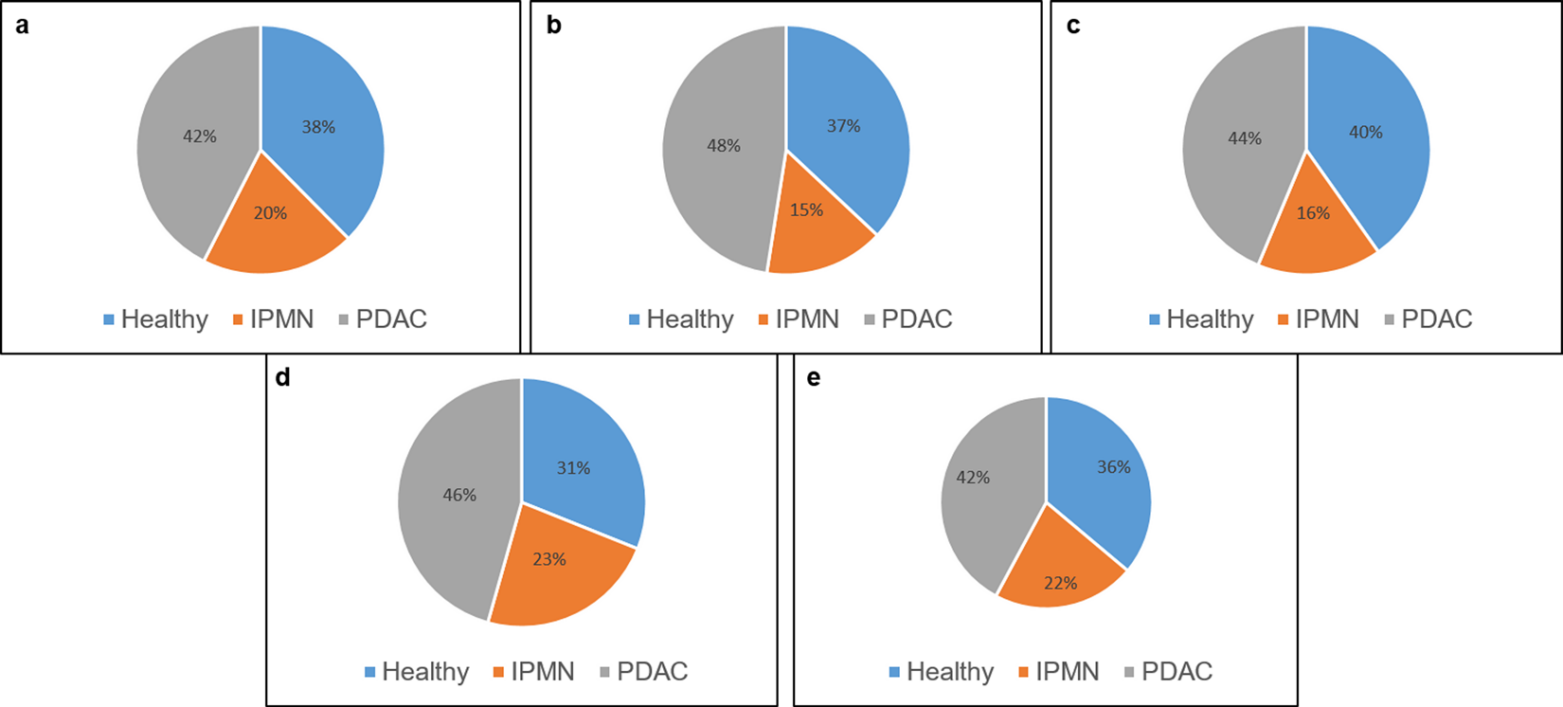
Pie charts depicting different RNAs in exosomes. The pie chart represents the mean percentage of each RNA biotype relative to mapped reads within exosomes obtained from healthy, IPMN and PDAC serum. mRNA (**a**), piRNA (**b**), tRNA (**c**), miRNA (**d**) and lincRNA (**e**).

Based on the mapped sequence read counts, the RNA content of different serum exosomes was quantified. The whole summary of reads mapping to gene annotation types are depicted in supplementary (Fig. S5). As illustrated in the pie chart graphs, serum exosomes contained very diverse RNA ‘cargo’. The following total gene types with varying abundance were present in exosomes from individual serum types:(mRNA (20,596), (lincRNA, 8660), ribosomal RNAs (rRNAs,3275), piwi interacting RNAs (piRNA, 2298), microRNAs (miRNA,1521) and transfer RNAs (tRNA/tRNA-like, 391). Also, other contents such as antisense elements to exons, introns, and small lncRNA, small Cajal body-specific RNAs (scaRNAs) including unannotated genes were also present in exosomes. Pie charts depicting the percentage of various RNAs between different serum exosomes are shown in Fig. 3. Overall, the abundance of these RNAs in exosomes are relatively high in PDAC serum compared to healthy and IPMN conditions. We will further focus only on three types of RNAs in this study (mRNA, piRNA and tRNA). Theoretically, it has been indicated that each exosome can accommodate 70–25,000 small RNA or protein molecules^27^. Given the size and high concentration of exosomes present in blood (10^10^–10^12^ particles per ml of blood)^31,36^ it is not surprising that exosomes could hold a variety of biological molecules within its boundaries and be capable of evoking considerable biological effects *in vivo*.

### Differentially expressed genes in serum exosomes

Total differentially expressed genes in exosomes between the serum types are shown as volcano plots in supplementary (Fig. S6). Overall, several genes were significantly up or down regulated in IPMN and PDAC compared to healthy serum exosomes. However, such differential expression of genes between two different healthy samples was very minimal. Often exosome studies involve reporting miRNA as biomarker candidates for cancer detection including that of the pancreas^37^. Other non-coding RNA types such as piRNA and tRNA are underrepresented and should be considered as an important source for differentiating the disease conditions independently or in combination with other RNA types. The ten differentially expressed mRNAs, piRNAs, and five tRNAs in different serum samples are listed in Tables 1–3. Heatmaps indicating the differences in various RNA expression are depicted in supplementary (Fig. S7). RNA-Seq raw data pertained to the depicted genes are shown in supplementary (File S1).

**Table 1:** RNA representation in serum derived exosomes. Transcripts organized by RNA types, mRNA, (1) piRNA (2) and tRNA (3). For each differentially expressed RNAs (FDR < 0.05), gene fold changes between groups are depicted. Raw data pertained to these different RNA types are shown in the supplementary excel file.

mRNA –Gene ID	IPMN/Healthy	PDAC/Healthy	PDAC/IPMN
MMP8	3.752300693	11.22053302	2.990307531
TBX3	2.495344919	6.110038834	2.448574859
PDX1	1.862313018	4.650163632	2.496982831
CTSL	0.940116753	9.621165889	10.23401175
SIGLEC15	1.139847435	3.261413452	2.861271914
IL32	1.126054868	0.538110379	0.477872255
SIGLEC11	2.197075703	0.387841497	0.176526233
DCN	1.039535435	0.587693174	0.565342127
HOXA5	0.531177671	0.066935876	0.126014101
KLRB1	0.82795046	0.308809226	0.372980318

**Table 2:** RNA representation in serum derived exosomes.

piRNA–Gene ID	IPMN/Healthy	PDAC/Healthy	PDAC/IPMN
hsa-piR-52959	0.737581056	48.80631338	66.17077944
hsa-piR-53108	0.737581056	48.70232593	66.029795
hsa-piR-30690	0.910145955	9.543237165	10.48539206
hsa-piR-54479	3.434405108	7.023957071	2.045174302
hsa-piR-56621	0.23389614	12.03718339	51.4637967
hsa-piR-54888	0.01989337	0.021437229	1.077606739
hsa-piR-42185	0.039457419	0.042519581	1.077606739
hsa-piR-46410	0.031030153	0.033438302	1.077606739
hsa-piR-58897	0.047468386	0.051152253	1.077606739
hsa-piR-43043	0.90149582	0.027407666	0.030402433

**Table 3:** RNA representation in serum derived exosomes.

tRNA–Gene ID	IPMN/Healthy	PDAC/Healthy	PDAC/IPMN
tRNA125-*Thr* CGT	17.23077001	24.37797911	1.414793366
tRNA21- *Ser* TGA	2.930639525	19.74772768	6.738368027
tRNA15-*Cys* GCA	1.657407286	2.041150703	1.231532357
tRNA55-Ile-TAT	0.56605197	0.149507902	0.264123985
tRNA5-*ILE* TAT	0.260427893	0.331645749	1.273464778

Among the different mRNA transcripts, *MMP8* was high in PDAC exosomes. MMP8, a member of the matrix metalloproteinase family, has been implicated in several cancer types and reported to have conflicting roles in cancer as a promoter and suppressor of metastasis^38^. However, the role of MMP8 in pancreatic disease is less known. While MMP 8 has been implicated in acute pancreatitis^39^ its function in PDAC is unclear. A different study suggested that *MMP8* could also function as a predictive biomarker in serum for colorectal cancer^40^. Another coding transcript, *TBX3* is also highly represented in PDAC exosome compared to healthy and IPMN serum. TBX3 protein product suppress E-cadherin and enhances melanoma invasiveness^41^ and is also correlated with advanced stages of gastric cancer^42^. While these genes were studied previously in direct plasma or tissues, their presence in exosomes have not been reported. Interestingly, we also observed increased representation of the pancreatic and duodenal homeobox-1 (*PDX1*) transcript in IPMN and tumor exosomes. The protein product from this gene was reported to be involved in the transcriptional activation of insulin and has been implicated in the pathological and clinical features of invasive PDAC^43^. Conversely, we also observed that some of the coding transcripts such as *SIGLEC11*^44^ which is implicated in anti-inflammatory function and *DCN* which functions as a tumor suppressor and anti-metastatic protein^45^ were low in PDAC and IPMN exosomes. Also, *KLRB1* (CD161) transcript appears to be low in IPMN and PDAC serum. KLRB1 transcript has been reported to be suppressed in lung tumors and esophageal squamous cell carcinoma^46^ although the exact biological function of this protein is unclear.

Like the protein coding transcripts, the presence of other small ncRNAs such as piRNA and tRNA transcripts were also observed in the exosomes. Previously, these ncRNAs were studied in either pancreatic tissues^47^ or cells but not in exosomes. For instance, tRNAs have been shown to interact with MEK2 in pancreatic carcinoma cells and alter cell behavior^48^. Likewise, piRNAs may have tumorigenic or suppressive roles in cancer and are likely involved in regulation of DNA methylation^49^. While piRNAs have been reported in variety of cancers^49^, reports are sparsely available for pancreatic cancer. One study, however, indicated that *piR-017061* was downregulated in pancreatic cancer tissues^47^. We observed several piRNA transcripts increased or decreased in PDAC exosomes relative to healthy or IPMN conditions (Table 2). It is unclear regarding the significance of above mentioned ncRNAs in exosomes. However, their expression seems to vary in pathological conditions. We speculate that these RNAs could be exchanged between the exosome target cells and may have functional significance.

### Validation of differentially expressed exosome mRNAs by qRT-PCR analysis

**Figure 4:**
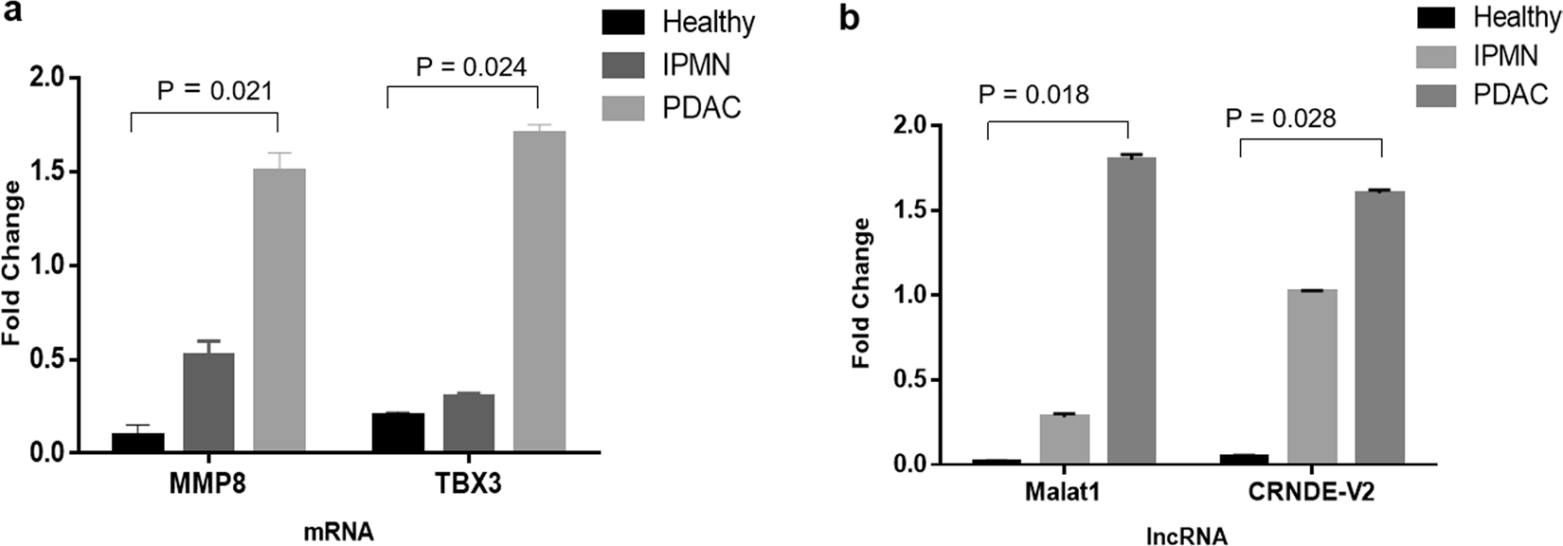
qRT-PCR analysis for RNA expression. Gene expression for MMP8 and TBX3 (**a**) including malat-1 and CRNDE lncRNA (**b**) were analyzed by qRT-PCR in the same serum exosomes used in EXO-NGS studies. Two biological replicates were used for each normal, IPMN and PDAC samples and the experiments were performed in duplicates. The dissociation curves for the qRT-PCR are shown in supplementary (Fig. 8).

To validate the EXO-NGS sequencing results, the expression of candidate genes *MMP8* and *TBX3* were analyzed in the serum exosomes under study. Consistent with NGS-EXO observations, both *MMP8* and *TBX3* were higher in tumor exosomes compared to healthy or IPMN samples with C_t_ values ranging from 27.6–30.8 with a median C_t_ of 29.2 (*MMP8*), and C_t_ values (28.0–31.7) with a median C_t_ of 29.8 (*TBX3*) (Fig. 4a).

Both lncRNAs *malat1 and CRNDE* have been reported to be increased in pancreatic cancer cells or tissues^50,51^. However, their presence in exosomes are less known. Due to limitation in analysis of gene size during EXO-NGS, lncRNA analysis in exosomes were conducted by qPCR analysis in exosomes isolated directly using the serum samples under investigation. The differences in the expression of lncRNAs *malat1* and *CRNDE* in serum exosomes are depicted in (Fig. 4b). Both lncRNAs were expressed higher in PDAC or IPMN vs healthy samples. The C_t_ values ranges were between 27 and 31 with a median C_t_ of 28.7 for *malat1* and C_t_ values between 29–32 with a median C_t_ of 30.5 for CRNDE. The dissociation curves corresponding to each gene is depicted in supplementary (Fig. S8).

While our studies indicate differences in various RNAs between serum types, the presence of exosome RNAs could be differentially regulated in pancreatic tumor subtypes. For instance, previous RNA sequence analysis in tissues from different pancreatic tumor subtypes^52^ which varied in their neoplastic cellularity^53^, indicated that an individual mRNA could be differentially regulated (up or down) within these subtypes. Therefore, it is reasonable to anticipate that exosomes and their components representing different cellular origins could likely mimic these changes. For instance, as observed in this study the exosome mRNA transcripts PDX1 and *CTSL (CTSL1)* both were found to be high in PDAC. Previous studies^52^ indicated the presence of these genes in pancreatic tumor tissues but high in progenitor and immunogenic subtypes compared to the basal type. Alternately, the *DCN* transcript which we observed to be low in PDAC serum exosomes was also absent or decreased in most tumor subtypes except immunogenic subtype. The mRNA distribution of these genes in PDAC serum exosomes from the present study, compared to tumor tissues of different subtypes reported earlier^52^ are depicted in Table 4.

**Table 4:** Gene expression comparison between exosomes and tumor subtypes. The transcripts expressed in exosomes of PDAC serum in the current study was compared with previously reported results obtained in tissues from different PDAC tumor subtypes. The data for PDAC tumor subtypes* are from ref. ^52^. The transcripts in exosomes appear to indicate the most likely PDAC tumor subtypes a particular gene may be up or down regulated.

mRNA –Gene ID	PDAC serum exosomes	PDAC Tumor subtypes*			
		Basal	Classical/Progenitor	ADEX	Immunogenic
PDX1	Up	Down	Up	Up	Down
CTSL	Up	None	Down	None	Up
DCN	Down	None	Down	None	Up

In the current study, we were able to investigate the RNAs in the patient serum exosomes, however, a limitation in this study is the exosome isolation technique. While various methods have been reported in the literature regarding exosome isolation^54^, each has its own advantages and disadvantages. Thus far, the established methods appear to lack robustness in obtaining pure exosome population. However, a previous report indicate that both ultracentrifugation and commercially available total exosome isolation reagents have been shown to recover exosomes in a comparable manner^55^. Another study, indicated that techniques such as precipitation (comparable to the method used in our studies) and membrane affinity are suitable for miRNA discovery in exosomes^4^. Overall, challenges in isolating exosomes include reproducibility and consistency between techniques. Both technical (e.g. sample collection) and biological (e.g. ideal matched control samples) challenges need to be considered to develop a reliable method for exosome analysis. Going forward, a standard and reproducible exosome enrichment method is necessary for its use in downstream clinical applications.

We identified several RNA types in serum exosomes, which may have the potential to be biomarkers and translatable for clinical applications to detect pancreatic disease early and differentiate among subtypes. Exosome RNA transcripts identified in this study such as *PDX1*, *CTSL and DCN* are reported to be variably expressed in different pancreatic tumor subtypes^52^ and could form the basis for investigating further in precursor lesions such as IPMN. Further validation of these biomolecules needs screening for more specific disease category and larger sample cohorts. Investigations of these biomolecules as markers should also consider biological diversity between patients. Also, any specific and abundantly expressed RNA transcripts in exosomes should exist within detectable levels for further validation as biomarkers in larger cohorts using routinely used technology platforms (e.g. qRT-PCR).

## Conclusion

Our studies indicate the presence of exosome vesicles of varying size in different serum samples. While NTA method may be used in analyzing exosomes, additional methods such as EM will be necessary to analyze exosomes. Unlike NTA, EM not only allows to visualize exosome morphology but enables to measure even small size vesicles below 40 nm. By performing EXO-NGS analysis we identified several RNA transcripts which have not been previously reported in exosomes associated with different pancreatic conditions. While most previous RNA studies have been done with pancreatic tumor cells or tissues, liquid biopsy sources such as exosomes would facilitate the noninvasive discovery of useful biomarkers for early detection. The presence of RNAs such a piRNA and tRNA, hitherto sparsely reported in pancreatic exosomes, suggested the need for further studies to gain insights into their biological effects on target cells. While our studies involve limited sample analysis, validation in large prospective cohorts which include more cases of premalignant lesions will be extremely useful in detecting the early disease. Our future studies are directed toward such validation efforts in patients to identify prognostic or predictive biomarkers using RNA signatures.

## Methods

### Collection of serum samples

After approval of the Institutional Review Board (IRB), Office of Research, University of Missouri, and informed consent from the human subjects for study participation, blood samples without anticoagulants from healthy individuals, intraductal papillary mucinous neoplasms (IPMN) and PDAC (stage III-IV) were collected. An hour after the blood collection, serum was separated by centrifugation (2300 rpm, 15 min) and used immediately for isolation of exosomes or stored at −80 °C for long term. If stored, 500 μl of serum was centrifuged at 1500 × g for 5 min to remove residual cells and debris. The supernatant was transferred to a new 1.5 ml Eppendorf tube before exosome isolation. All methods were performed in accordance with relevant guidelines and regulations.

### Exosome isolation from serum

ExoQuick Ultra (System Biosciences, Palo Alto, CA) was added to the supernatant from the above step at a 1:4 ratio, mixed gently, and incubated for 30 min at 4 °C. After the incubation, the admixture was centrifuged at 1500 × g for 30 min to recover exosomes for total RNA isolation. For gel purification, serum was diluted with PBS (two-fold) and processed using qEV size exclusion columns (IZON, Medford, MA) according to manufacturer’s instructions.

### Exosome particle size analysis

Exosomes obtained from serum were diluted in PBS (dilution depending on the concentration of exosomes) and subsequently analyzed using a NanoSight NS300. The instrument is equipped with a 405 nm laser (NanoSight, Amesbury, UK), and an electron multiplying charge‐coupled device (EMCCD) was used to determine the histogram of particle size by tracking the Brownian motion of single particles at 25 °C for size and concentration determination. Videos were recorded for 60 seconds during which the movement of the nanoparticles were tracked by NTA software (version 2.3, NanoSight) with low refractive index corresponding to serum derived vesicles. The Stokes-Einstein equation was employed to determine the size distribution and number of particles within the sample.

### Transmission electron microscopy

Exosomes isolated from the serum were processed for transmission electron microscopy (TEM) and negative staining. All specimen preparation was performed at the Electron Microscopy Core Facility, University of Missouri. Exosomes were suspended in PBS (pH 7.2), and placed on a negatively charged carbon coated copper grid and allowed to settle at room temperature for 20 minutes. Excess sample was removed by wicking with filter paper, then fixed for two minutes in 2% paraformaldehyde and, 2% glutaraldehyde in 0.05 M phosphate buffer (pH 7.0) solution. Grids were rinsed with three consecutive washes of distilled water and negative staining was performed by placing 5 μL of Nano Tungsten (Nanoprobes, Inc.) on each grid for two minutes prior to wicking to dryness with filter paper and allowed to dry at room temperature. Images were acquired with a JEOL JEM 1400 transmission electron microscope (JEOL, Peabody, MA) at 120 kV on a Gatan Ultrascan 1000 CCD (Gatan, Inc, Pleasanton, CA).

### Total RNA isolation from exosomes

Total RNA from exosomes was isolated using the SeraMir Exosome RNA Purification kit (System Biosciences) according to the manufacturer’s instructions. Subsequently, to measure RNA concentration and integrity of each sample, 1 μl of the final RNA eluate was used for measurement of small RNA concentration by Agilent Bioanalyzer Small RNA Assay using Bioanalyzer 2100 Expert instrument (Agilent Technologies, Santa Clara, CA).

### Immunoblotting

Exosomes isolated from serum were suspended in 50 μl M-PER reagent (Thermo Scientific) with HALT protease inhibitor cocktail (Thermo Scientific). After determining the protein concentration using Bradford analysis, the samples were fractionated on a 4–12% gradient SDS-PAGE gels (Invitrogen, Carlsbad, CA). Subsequently, the gels were transferred to a nitrocellulose membrane (Bio-Rad, Hercules, CA). The membrane was incubated with anti-CD63 (H5C6) (Novus Biologicals, Centennial, CO) or anti-CD9 (D3H4P) antibodies (Cell Signaling Technology, Danvers, MA) overnight at 4 °C, followed by incubation with peroxidase conjugated anti-mouse/-rabbit antibody. The proteins were visualized using an Immobilon Forte Western HRP blotting substrate (Millipore, Burlington, MA).

### NGS Library generation and sequencing

Small RNA libraries were constructed with the Clean Tag Small RNA Library Preparation Kit (TriLink, Cat# L-3206) according to the manufacturer’s protocol. The final purified library was quantified with High Sensitivity DNA Reagents (Agilent Technologies, PO# G2933-85004) and High Sensitivity DNA Chips (Agilent Technologies, PO# 5067–4626). The libraries were pooled, and the 140 bp to 300 bp region was size selected on an 8% TBE gel (Invitrogen, Life Technologies). The size selected library was quantified with High Sensitivity DNA 1000 Screen Tape (Agilent Technologies, PO # 5067–5584), High Sensitivity D1000 reagents (Agilent Technologies, PO# 5067–5585), and the TailorMix HT1 qPCR assay (SeqMatic, Cat# TM-505), followed by a NextSeq High Output single-end sequencing run at SR75 using NextSeq. 500/550 High Output v2 kit (Cat #FC-404-2005, Illumina, San Diego, CA) according to the manufacturer’s instructions.

### Bioinformatic analysis

#### Raw reads filtering

The Exosome RNA-seq analysis initiates with a data quality check of the input sequence using FastQC, an open-source quality control (QC) tool for high-throughput sequence data^56^. FastQC runs analyses of the uploaded raw sequence reads that reveals the quality of the data and inform the subsequent preprocessing steps in the analysis. Following the initial assessment, Bowtie2^57^ was used to map the spike-in DNA before the trimming and filtering steps where RNA-seq reads are preprocessed to improve the quality of data input for read mapping. The open-source tools used for trimming of adapters are FastqMcf^56^ and Cutadapt^58^, while PRINSEQ^59^ was used in the quality filtering step.

#### Reads mapping

Sequencing adapters were trimmed from reads, and filtered for quality and length in the preprocessing steps. FastQC is then re-run to analyze the trimmed reads, allowing for a before and after comparison. The processed set of input data was used in the subsequent read mapping step. The improved set of sequence reads are merged, if needed, using SeqPrep^60^ then mapped to the reference genome assembly (GRCh37-hg19) using Bowtie2^57^. Using the open-source software BEDTools^61^ and SAMtools^62^, read alignment and read coverage tracks are generated and deployed to the genome browser.

#### RNA abundance analysis

Abundance determination and differential expression analysis was performed by DEseq^63^. Abundance levels for ncRNAs (miRNAs, tRNAs, rRNAs, lincRNAs, piRNAs, snoRNAs), antisense transcripts, coding genes and repeat elements (LTR, LINE, SINE, and tandem repeats) were determined. A summary of reads overlapping each of these annotations in the reference genome is created using SAM tools.

#### cDNA synthesis and quantitative real-time PCR (qRT-PCR)

Exosome RNA was converted to cDNA using a High Capacity Reverse Transcription kit (Applied Biosystems, Foster City, CA) with RNAse inhibitor treatment according to manufacturer’s protocol. qRT-PCR was performed using SYBR Green assay for exosome mRNA expression (n = 3 each sample) of matrix metalloproteinase-8 (MMP-8), TBX3 and long non-coding RNAs, malat-1 and CRNDE. β-actin was used as internal control. Relative quantification (fold change) was determined using 2^−ΔΔCt^ method. Following primers were used in this study: MMP8, F-CCTGGTGCCTTGATGTA, R- GGTCCAGTAGGTTGGATAG; TBX3, F- GTGACTGCATACCAGAAT, R-TTCTCTTCGGCCATTTC; Malat1, F-GTTCTGATCCCGCTGCTATT, R-TCCTCAACACTCAGCCTTTATC; CRNDE, F- GTTGTCACGCAGAAGAAG, R-TCCTATACCTTGGCTAAACA. Two replicate serum samples each from healthy, IPMN and PDAC were used. Analysis was done in duplicates.

#### Statistical analyses

Differences in gene expression levels were analyzed using either a paired t-test or nonparametric two-tailed Mann Whitney –U test. P value < 0.05 was considered significant.

## Supplementary information

Supplementary figures

## Data Availability

The data pertained to this manuscript are available to readers upon publication. Certain materials as a part of this data but not associated with this manuscript may not be shared due to either ongoing analysis or publication restrictions.
