# Author Correction: RNA cargos in extracellular vesicles derived from blood serum in pancreas associated conditions

**DOI:** 10.1038/s41598-020-66766-4

**Published:** 2020-06-16

**Authors:** Senthil R. Kumar, Eric T. Kimchi, Yariswamy Manjunath, Saivaroon Gajagowni, Alexei J. Stuckel, Jussuf T. Kaifi

**Affiliations:** 1 Veterinary Medicine & Surgery, College of Veterinary Medicine, University of Missouri, Columbia, MO 65211 USA; 2 Department of Surgery, School of Medicine, University of Missouri, Columbia, MO 65212 USA; 3 Harry S. Truman Veterans Hospital, 800 Hospital Drive, Columbia, MO 65212 USA; 4 Department of Medicine, Division of Gastroenterology and Hepatology, University of Missouri, Columbia, MO 65212 USA

Correction to: *Scientific Reports* 10.1038/s41598-020-59523-0, published online 18 February 2020

Jussuf T. Kaifi was inadvertently omitted from the author list in the original version of this Article. This has been corrected in the PDF and HTML versions of this Article, and in the accompanying Supplementary Information.

The Author Contributions section now reads:

S.R.K. conceived the study and wrote the manuscript. E.T.K. coordinated patients for this study. S.G. performed the experiments along with S.R.K. Y.M. along with E.T.K. had direct contact with the patients to collect blood samples and subsequent processing for downstream studies. S.R.K. and A.J.S. performed the bioinformatic analysis. J.T.K. as the responsible IRB primary investigator coordinated patient recruitment and subjects’ consenting, blood sample acquisition and processing/analysis in the laboratory. All authors reviewed the manuscript.

Additionally, the original version of this Article omitted an affiliation for Alexei J. Stuckel. The correct affiliations for Alexei J. Stuckel are listed below:

Harry S. Truman Veterans Hospital, 800 Hospital Drive, Columbia, MO 65212, USA

Department of Medicine, Division of Gastroenterology and Hepatology; University of Missouri, Columbia, MO 65212, USA

This has also now been corrected in the HTML and PDF versions of this Article, and in the accompanying Supplementary Information.

