# Supplementary material for: RNA cargos in extracellular vesicles derived from blood serum in pancreas associated conditions
Source: Sci Rep. 2020 Feb 18;10:2800. doi: 10.1038/s41598-020-59523-0 (PMC7028741; doi:10.1038/s41598-020-59523-0)
Supplement: Supplementary file 1 — Supplementary figures [file 41598_2020_59523_MOESM1_ESM.docx]

**RNA cargos in extracellular vesicles derived from blood serum in pancreas associated conditions**

Senthil R. Kumar ^1,2,3*^, Eric T. Kimchi^2,3^, Yariswamy Manjunath^2,3^, Saivaroon Gajagowni^1^, Alexei J. Stuckel^3,4^, Jussuf T. Kaifi ^2,3^

^1^Veterinary Medicine & Surgery, College of Veterinary Medicine, University of Missouri, Columbia, MO 65211, USA; ^2^ Department of Surgery, School of Medicine, University of Missouri, Columbia, MO 65212, USA; ^3^Harry S. Truman Veterans Hospital, 800 Hospital Drive, Columbia, MO 65212, USA; ^4^Department of Medicine, Division of Gastroenterology and Hepatology; University of Missouri, Columbia, MO 65212, USA.

**
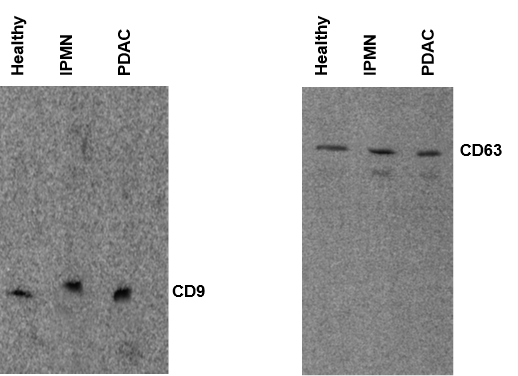
**

**Figure S1.** Immunoblots for exosome marker proteins. Exosome markers CD63 and CD9 proteins were detected using respective antibodies in different serum. CD63 ~95 kDa, CD9 ~ 24 kDa.


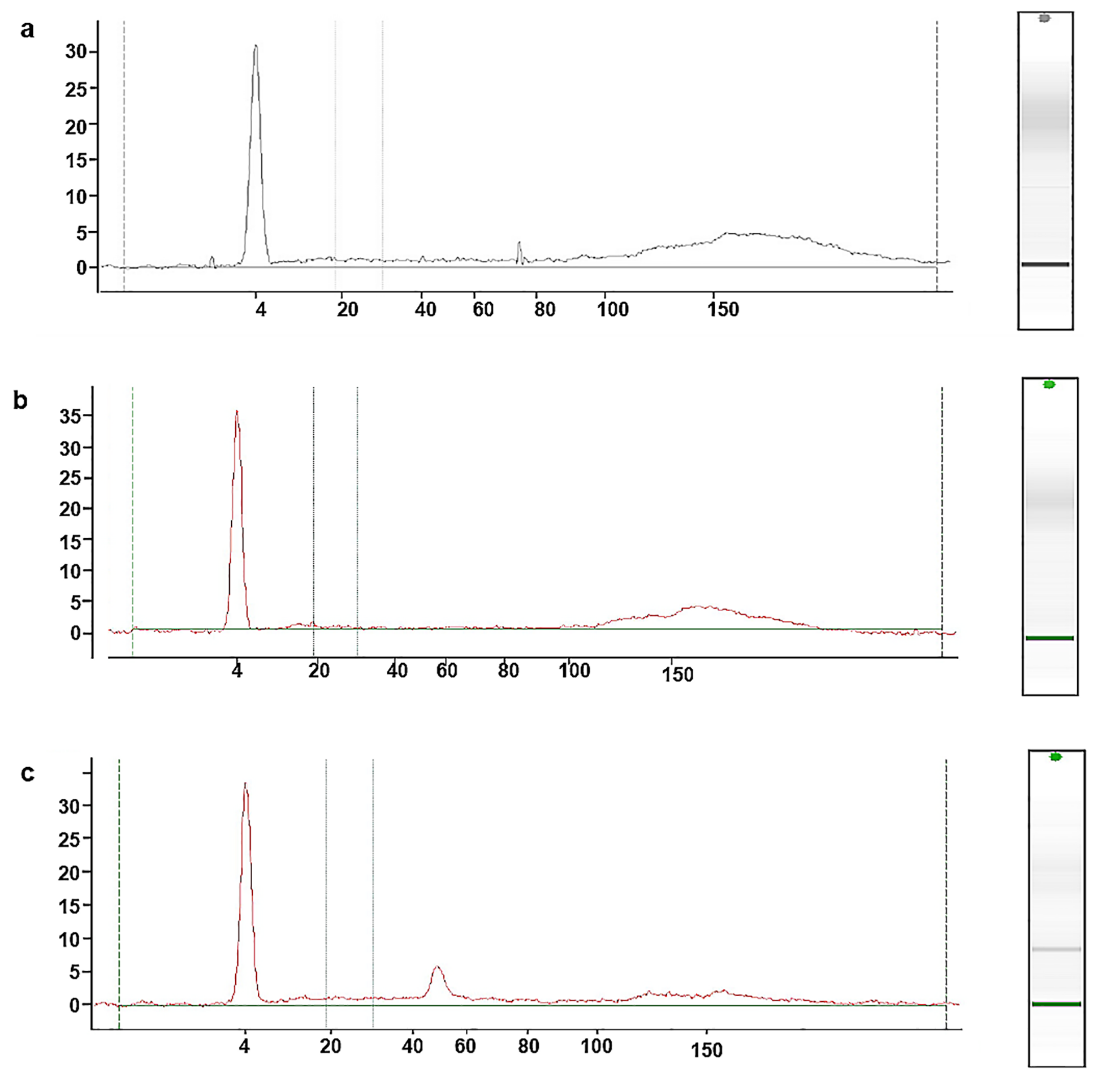


**Figure S2**. RNA analysis by Bioanalyzer. Total RNA was isolated from the exosomes and RNA (6-150 nucleotides) was analyzed with PicoChip using Agilent 2100 Bioanalyzer; a) Healthy, b) IPMN, c) PDAC.

**
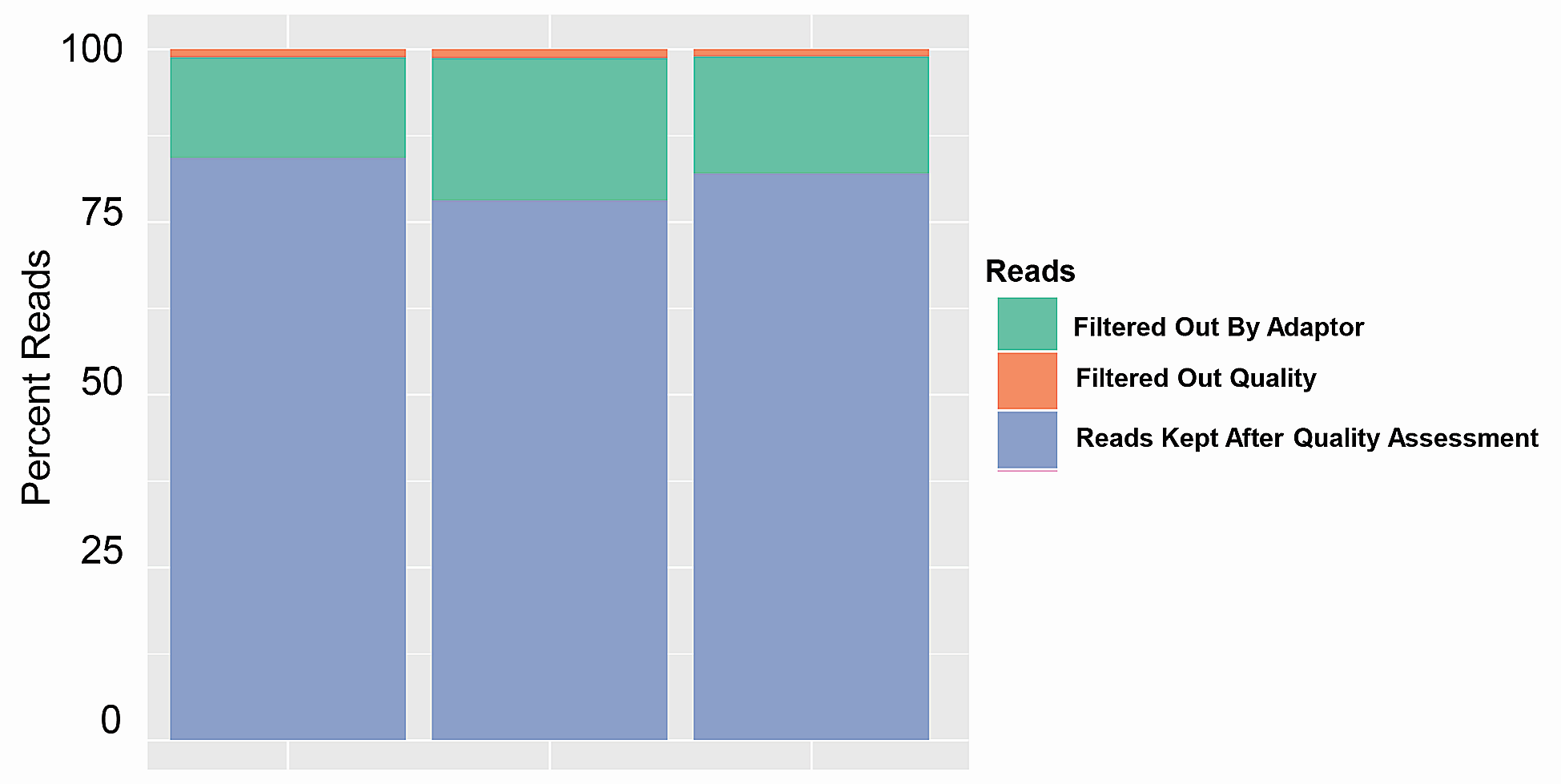
**

**Figure S3.** Sequence reads for quality. Number of reads before and after quality assessment and filtering in different samples and indicated by color codes.

**
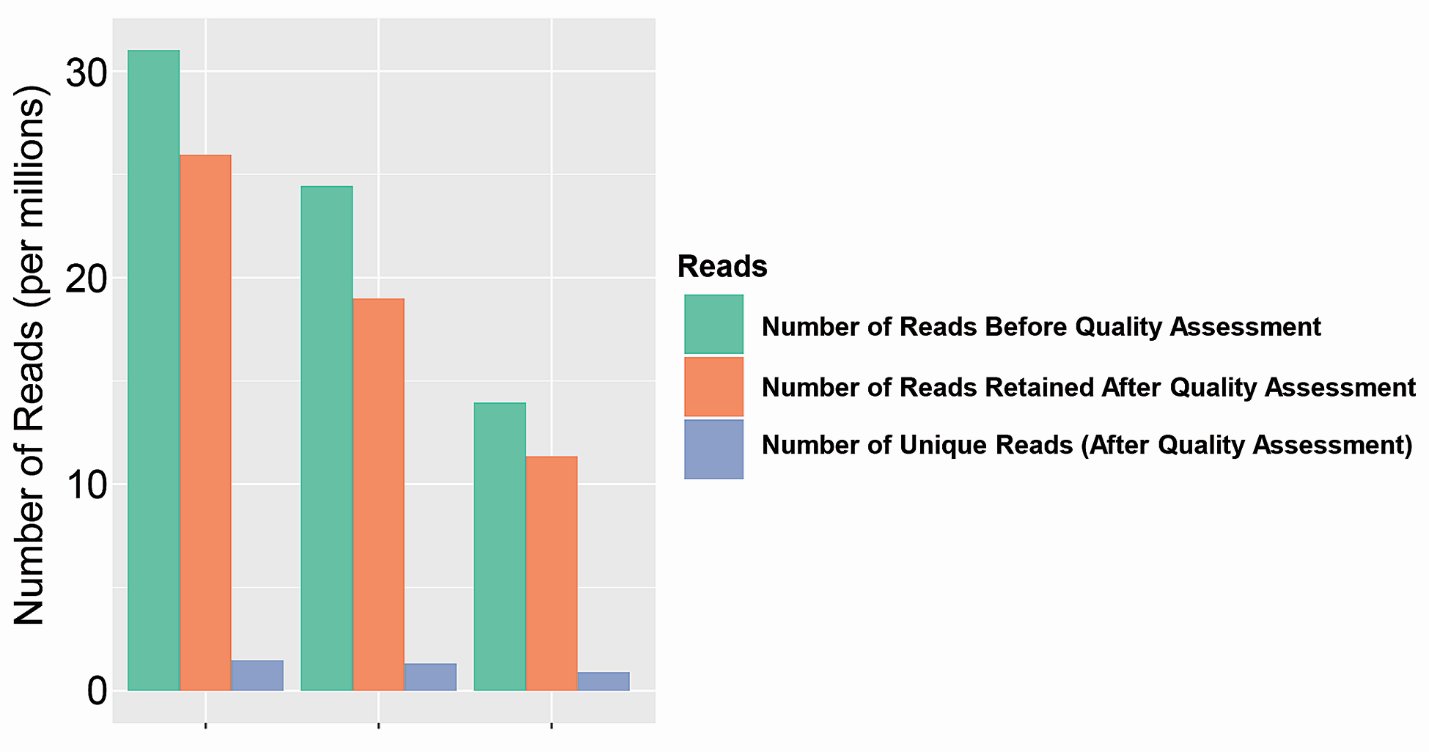
**

**Figure S4.** Sequence read quality assessment. Quality assessment (QA) results are shown. Reads retained after QA are shown in blue. Reads that were discarded after adapter trimming or due to poor quality are shown in green and orange, respectively.

**
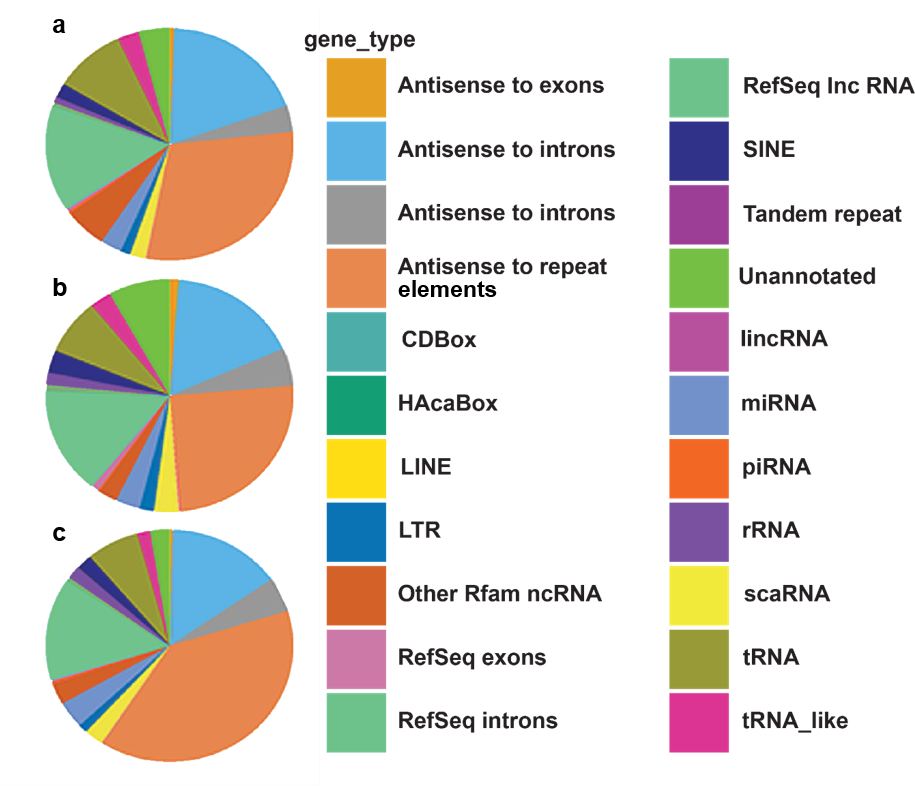
**

**Figure S5.** Pie charts depicting various annotation types in serum exosomes. The pie chart is a representative of reads derived from different RNAs and other annotation categories in serum exosomes. IPMN (a), PDAC (b), Healthy (c).

**
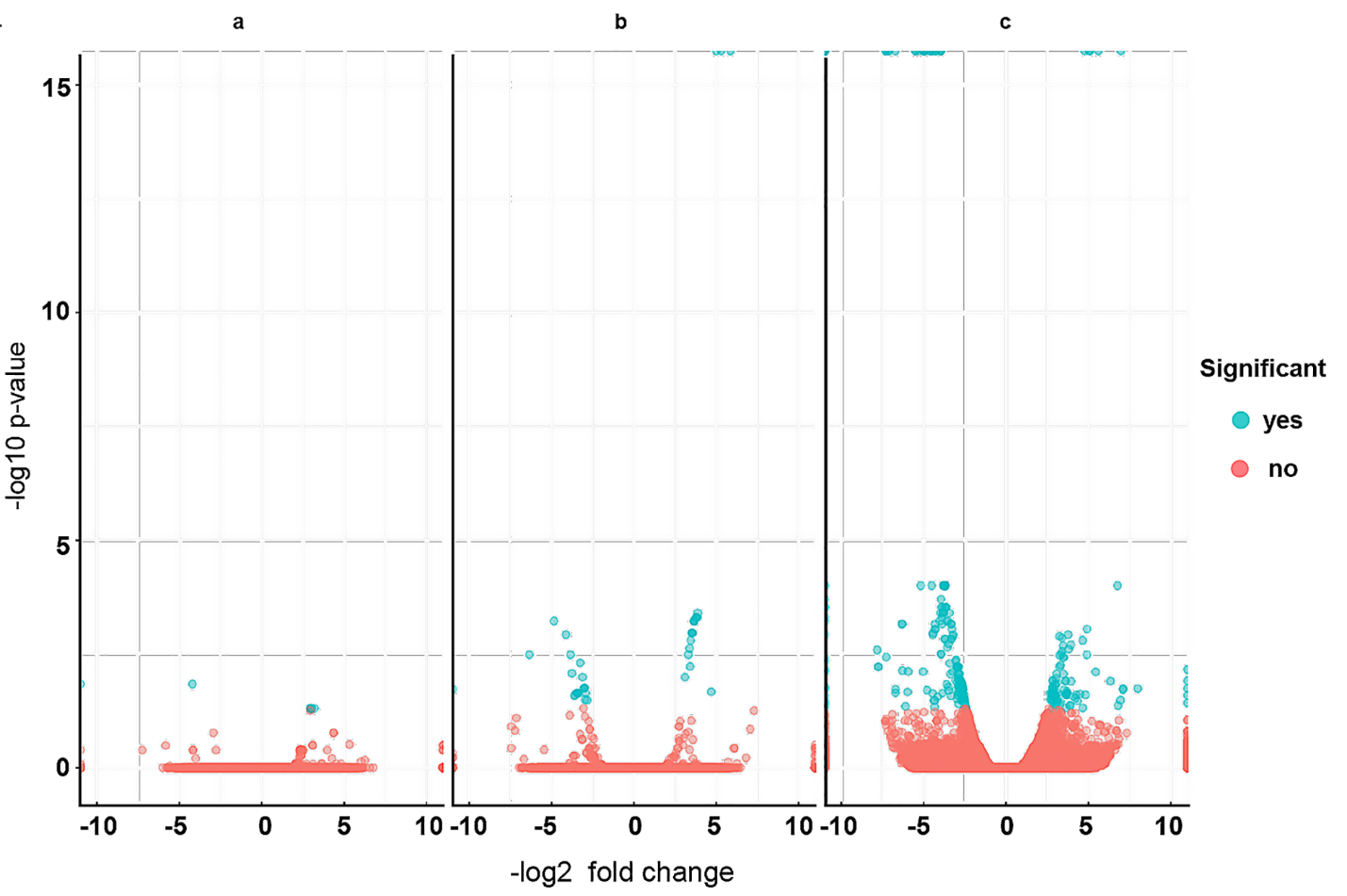
**

**Figure S6.** Volcano plots depicting gene fold changes in serum exosomes. Fold-change (log2) versus significance (-log10 p-value) for each gene is shown between serum samples. a. Normal vs normal serum; b. Normal vs IPMN; c. Normal vs PDAC. Significant genes (FDR < 0.05) are in blue.

**
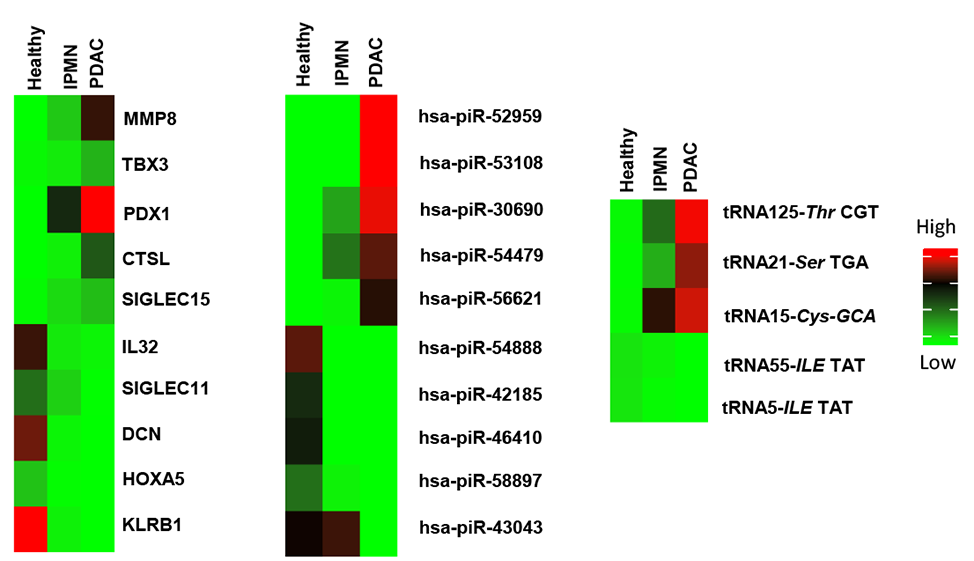
**

**Figure S7.** Heatmaps of differentially expressed genes. Differences in the expression of mRNA, piRNA and tRNA between samples are calculated using DESeq analysis. Differentially expressed genes are identified based on a model using the negative binomial distribution and visual representation of the analysis is provided as heatmap view.


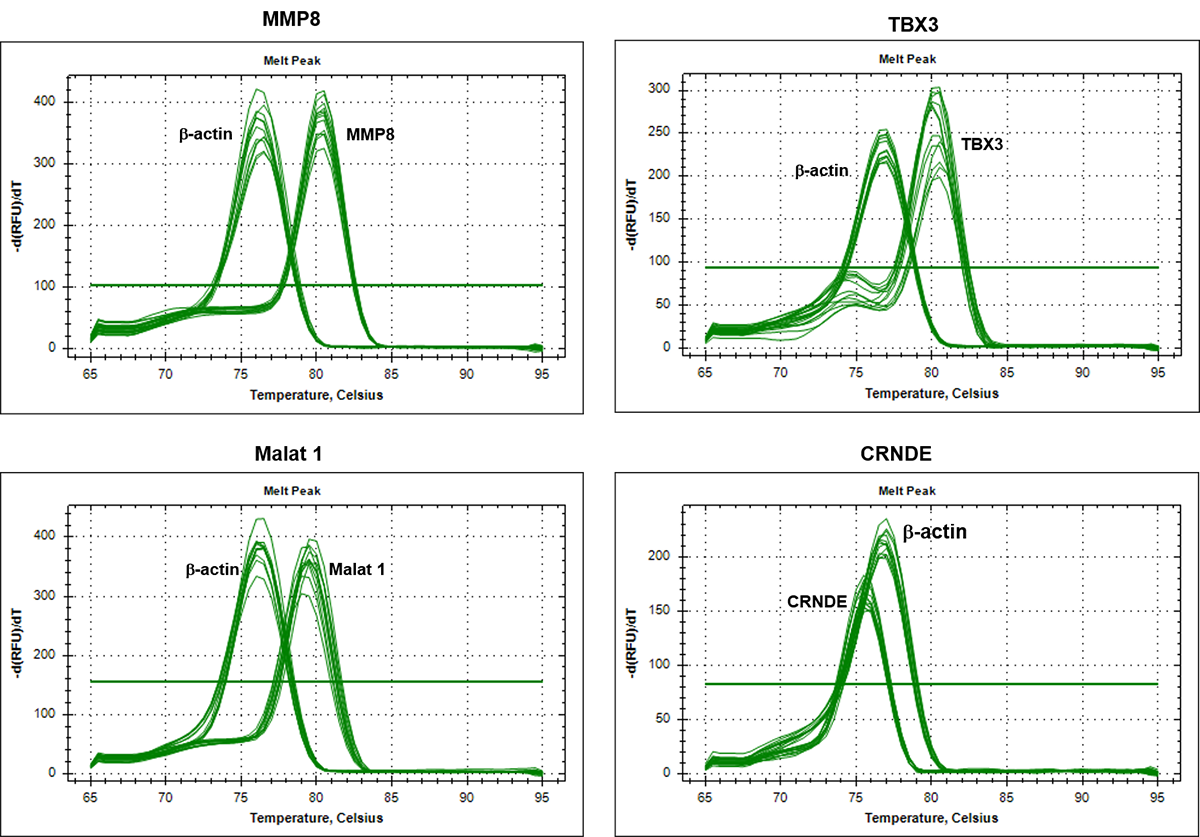


**Figure S8**. Dissociation curves for qRT-PCR gene analysis. Dissociation curves for different target genes (MMP8, TBX3, Malat 1 & CRNDE) and a reference gene (β-actin) (see Fig 4) in the serum exosomes.
